# Identifying Household Diabetes Risk for Family Diabetes Prevention Using Electronic Health Records

**DOI:** 10.1001/jamanetworkopen.2025.51823

**Published:** 2026-01-13

**Authors:** Tainayah W. Thomas, Holly Finertie, Arjun Silverberg, Maher Yassin, James E. Patino, Lisa G. Rosas, June Tester, Luis A. Rodriguez, O. Kenrik Duru, Julie A. Schmittdiel

**Affiliations:** 1 Department of Epidemiology and Population Health, Stanford University School of Medicine, Stanford, California; 2 Division of Research, Kaiser Permanente Northern California, Pleasanton; 3 University of California, Los Angeles; 4 Division of Primary Care and Population Health, Department of Medicine, Stanford University School of Medicine, Palo Alto, California; 5 Department of Pediatrics, University of California, San Francisco; 6 Department of Health Systems Science, Kaiser Permanente Bernard J. Tyson School of Medicine, Pasadena, California; 7 Department of Epidemiology and Biostatistics, University of California, San Francisco; 8 Division of General Internal Medicine-Health Services Research, David Geffen School of Medicine at UCLA, Los Angeles, California

## Abstract

This cohort study uses electronic health record (EHR) data to evaluate diabetes risk factors among household members of adults with prediabetes.

## Introduction

Diabetes is a transgenerational disease that can affect children, parents, and grandparents.^1^ Because diabetes risk clusters in families,^2^ who often receive care in the same health system, electronic health records (EHRs) could be used to identify and screen coresiding family members to improve diabetes prevention efforts. This study used health system EHR data to identify household members of adults with prediabetes and evaluate their diabetes risk factors, by age.

## Methods

We identified an index cohort of adults with prediabetes (fasting plasma glucose, 100-125 mg/dL [to convert glucose to millimoles per liter, multiply by 0.0555] or hemoglobin A_1c_, 5.7%-6.4% [39-46 mmol/mol; to convert to proportion of total hemoglobin, multiply by 0.01]) and body mass index (BMI) of 25 or more (calculated as weight in kilograms divided by height in meters squared) within 1 year prior to January 1, 2023, through December 31, 2023. The index date was the earliest date in 2023 on which patients met inclusion criteria. We excluded patients with diabetes, end-stage kidney disease, or less than 12 months’ health system membership within 1 year before index date. We identified coinsured members living with the index member at index date through the index member’s subscriber medical record numbers and address history. This study was approved by the UCLA institutional review board, and a waiver of informed consent was obtained due to the minimal risk to human participants. This observational cohort study followed the STROBE reporting guideline for cohort studies.

Diabetes risk factors were calculated for those aged 10 years or older. For adults, risk factors were defined as BMI of 25 or more or a history of gestational diabetes, hypertension, dyslipidemia, or cardiovascular disease or prediabetes or diabetes. For children aged 10 to 17 years, risk factors were age- and sex-specific^3^ overweight or obesity or prediabetes or diabetes. Descriptive statistics were output for the index cohort and household members. Patients self-reported race and ethnicity. Analyses were performed in SAS, version 9.4 and R, version 4.3.1. Detailed methods are provided in the eMethods in Supplement 1.^4^

## Results

The index cohort included 356 626 adults with prediabetes (mean [SD] age, 50.5 [12.0] years; 51.7% women and 48.3% men; 27.9% Asian American, 9.1% Black or African American, 1.1% Hawaiian or Pacific Islander, 26.6% Hispanic or Latino, 0.4% Native American or American Indian, 30.1% White, 0.8% multiracial, and 4.1% unknown (Table 1). Obesity was present in 58.5% of the index cohort. Household composition varied (48.1% 1-person households and 51.9% multiresident households). A total of 75.9% of multiresident households had at least 1 additional household member with diabetes risk factors.

**Table 1.** Baseline Demographic, Clinical, and Household Characteristics of Adults With Prediabetes

Characteristic	Adults, No. (%)		
	Overall (N = 356 626)	One-person household (n = 171 615)	Multiperson household (n = 185 011)
Demographic			
Age, mean (SD), y	50.5 (12.0)	50.7 (13.4)	50.4 (10.6)
Sex			
Female	184 297 (51.7)	95 081 (55.4)	89 216 (48.2)
Male	172 256 (48.3)	76 491 (44.6)	95 765 (51.8)
Other or unknown	73 (0.02)	43 (0.03)	30 (0.02)
Race and ethnicity			
Asian American	99 322 (27.9)	41 982 (24.5)	57 340 (31.0)
Black or African American	32 298 (9.1)	19 882 (11.6)	12 416 (6.7)
Hawaiian or Pacific Islander	4017 (1.1)	1827 (1.1)	2190 (1.2)
Hispanic or Latino	94 728 (26.6)	46 997 (27.4)	47 731 (25.8)
Native American or American Indian	1422 (0.4)	685 (0.4)	737 (0.4)
White	107 517 (30.1)	51 623 (30.1)	55 894 (30.2)
Multiracial	2729 (0.8)	1393 (0.8)	1336 (0.7)
Unknown	14 593 (4.1)	7226 (4.2)	7367 (4.0)
Insurance type			
Commercial	321 654 (90.2)	137 805 (80.3)	183 849 (99.4)
Medi-Cal	34 972 (9.8)	33 810 (19.7)	1162 (0.6)
Membership type			
Adult subscriber	285 616 (80.1)	166 895 (97.2)	118 721 (64.2)
Adult dependent	67 220 (18.8)	4170 (2.4)	63 050 (34.1)
Child or grandchild^a^	3610 (1.0)	519 (0.3)	3091 (1.7)
Disabled dependent	143 (0.04)	22 (0.01)	121 (0.1)
Other dependent^b^	37 (0.01)	9 (0.005)	28 (0.02)
BMI^c^	32.0 (6.6)	32.4 (6.9)	31.5 (6.2)
Overweight	148 142 (41.5)	68 965 (40.2)	79 177 (42.8)
Obese grade 1	111 892 (31.4)	52 244 (30.4)	59 648 (32.2)
Obese grade 2	54 433 (15.3)	27 077 (15.8)	27 356 (14.8)
Obese grade 3	42 159 (11.8)	23 329 (13.6)	18 830 (10.2)
Clinical			
HbA_1c_, mean (SD), %	5.9 (0.2)	5.9 (0.2)	5.9 (0.2)
Count of HbA_1c_ tests	340 363 (95.4)	164 116 (95.6)	176 247 (95.3)
FPG, mean (SD), mg/dL	106.1 (5.9)	106.2 (6.0)	106.0 (5.8)
Count of FPG tests	16 263 (4.6)	7499 (4.4)	8764 (4.7)
Household			
Household size (including index participant), No. of persons			
1	171 615 (48.1)	171 615 (100.0)	0
2	84 573 (23.7)	0	84 573 (45.7)
3	43 234 (12.1)	0	43 234 (23.34)
≥4	57 204 (16.0)	0	57 204 (30.9)
Household composition (not including index participant)			
Households with ≥1 household member <18 y	NA	NA	78 217 (42.3)
Households with ≥1 household member with diabetes risk factors	NA	NA	140 398 (75.9)

Abbreviations: BMI, body mass index (calculated as weight in kilograms divided by height in meters squared); FPG, fasting plasma glucose; HbA_1c_, hemoglobin A_1c_; NA, not applicable.

SI conversion factors: To convert HbA_1c_ to proportion of total hemoglobin, multiply by 0.01; and to convert glucose to millimoles per liter, multiply by 0.0555.

^a^ Adults with membership type child or grandchild include young adults covered as dependents on their parents’ or grandparents’ plans.

^b^ Other membership types include sponsored dependent and dependent of dependent.

^c^ BMI for all racial and ethnic groups except Asian American group: overweight, 25 to less than 30; obesity grade 1, 30 to less than 35; obesity grade 2, 35 to less than 40; and obesity grade 3, 40 or more. BMI for Asian American group: overweight, 23 to less than 27.5; obesity grade 1, 27.5 to less than 32.5; obesity grade 2, 32.5 to less than 37.5; and obesity grade 3, 37.5 or more. All index members had a BMI of 25 or higher (or ≥23 if Asian), which was required to enter the study.

We identified 364 563 coresiding household members (Table 2). The mean (SD) age was 41.5 (16.4) years for adult household members and 10.2 (4.8) years for child household members. Diabetes risk factors were identified in 64.6% of adults and 35.0% of children (aged 10-17 years); overweight or obesity was the most identified risk factor (adults, 54.9%; children, 34.8%). Abnormal blood glucose was present in 32.5% of adult household members, with 20.3% having prediabetes laboratory test results and 12.2% having evidence of diabetes. Prediabetes was most prevalent among Asian American adult household members (26.0%), and diabetes was most prevalent among Hawaiian or Pacific Islander adult household members (15.6%).

**Table 2.** Baseline Demographic and Clinical Characteristics of Individual Household Members of Adults With Prediabetes

Characteristic	Household members, No. (%)		
	Overall (N = 364 563)	Adult (≥18 y) (n = 238 247)	Child (<18 y) (n = 126 316)
Demographic			
Age, mean (SD), y	30.7 (20.2)	41.5 (16.4)	10.2 (4.8)
Sex			
Female	187 233 (51.4)	125 765 (52.8)	61 468 (48.7)
Male	177 257 (48.6)	112 431 (47.2)	64 826 (51.3)
Other or unknown	73 (0.02)	51 (0.02)	22 (0.02)
Race and ethnicity			
Asian American	106 645 (29.3)	68 315 (28.7)	38 330 (30.3)
Black	20 780 (5.7)	13 594 (5.7)	7186 (5.7)
Hawaiian or Pacific Islander	4501 (1.2)	2809 (1.2)	1692 (1.3)
Hispanic or Latino	95 891 (26.3)	62 016 (26.0)	33 875 (26.8)
Native American or American Indian	1353 (0.4)	968 (0.4)	385 (0.3)
White	102 034 (28.0)	74 068 (31.1)	27 966 (22.1)
Multiracial	6285 (1.7)	2587 (1.1)	3698 (2.9)
Unknown	27 074 (7.4)	13 890 (5.8)	13 184 (10.4)
Membership type^a^			
Adult subscriber	63 949 (17.5)	63 945 (26.8)	0
Adult dependent	96 858 (26.6)	96 851 (40.7)	0
Child or grandchild	202 813 (55.6)	76 675 (32.2)	126 138 (99.9)
Disabled dependent	811 (0.2)	727 (0.3)	84 (0.1)
Other dependent	132 (0.04)	49 (0.02)	94 (0.1)
BMI^b^	25.5 (7.3)	28.5 (6.6)	20.5 (5.5)
Normal	122 428 (34.1)	53 894 (22.6)	68 534 (56.6)
Overweight	78 126 (21.7)	59 971 (25.2)	18 155 (15.0)
Obese	93 953 (26.1)	70 804 (29.7)	23 149 (19.1)
Missing	64 809 (18.0)	53 578 (22.5)	11 231 (9.3)
Clinical			
HbA_1c_, mean (SD), %	5.9 (1.1)	6.0 (1.2)	5.5 (0.5)
Count of HbA_1c_ tests	154 288 (42.3)	137 058 (57.5)	17 230 (13.6)
FPG, mean (SD), mg/dL	101.3 (35.1)	102.3 (35.5)	87.8 (26.3)
Count of FPG tests	36 043 (9.9)	33 488 (14.1)	2555 (2.0)
No. of primary care visits			
0	87 625 (24.0)	69 132 (29.0)	18 493 (14.6)
1	98 157 (26.9)	60 943 (25.6)	37 214 (29.5)
≥2	178 781 (49.0)	108 172 (45.4)	70 609 (55.9)
No. of glucose screening visits			
0	205 859 (56.5)	97 443 (40.9)	108 416 (85.8)
1	85 171 (23.4)	70 469 (29.6)	14 702 (11.6)
≥2	73 533 (20.2)	70 335 (29.5)	3198 (2.5)
Diabetes risk factors^c^	179 449 (57.7)	153 996 (64.6)	25 453 (35.0)
Overweight or obese	156 101 (50.2)	130 775 (54.9)	25 326 (34.8)
Gestational diabetes	1219 (0.5)	1219 (0.5)	NA
Hypertension	45 278 (19.0)	45 278 (19.0)	NA
Dyslipidemia	38 416 (16.1)	38 416 (16.1)	NA
Cardiovascular disease	3848 (1.6)	3848 (1.6)	NA
Prediabetes laboratory result	48 297 (20.3)	48 297 (20.3)	3174 (4.4)
Evidence of diabetes	29 282 (9.4)	28 997 (12.2)	285 (0.4)

Abbreviations: BMI, body mass index (calculated as weight in kilograms divided by height in meters squared); FPG, fasting plasma glucose; HbA_1c_, hemoglobin A_1c_; NA, not applicable.

SI conversion factors: To convert HbA_1c_ to proportion of total hemoglobin, multiply by 0.01; and to convert glucose to millimoles per liter, multiply by 0.0555.

^a^ Adults with membership-type child or grandchild included young adults covered as dependents on their parents’ or grandparents’ plans. Other membership types include sponsored dependent and dependent of dependent.

^b^ BMI for all racial and ethnic groups except Asian American group: overweight, 25 to less than 30; obesity grade 1, 30 to less than 35; obesity grade 2, 35 to less than 40; and obesity grade 3, 40 or more. BMI for Asian American group: overweight, 23 to less than 27.5; obesity grade 1, 27.5 to less than 32.5; obesity grade 2, 32.5 to less than 37.5; and obesity grade 3, 37.5 or more. BMI reported only for children aged 2 years or older (n = 121 069).

^c^ Diabetes risk factors calculated only for children aged 10 to 17 years (n = 72 697).

## Discussion

In a cohort of patients with prediabetes and their household members, 75.9% of multiresident households had at least 1 additional household member with diabetes risk factors. Limitations include generalizability of EHR data to the general population, potentially incomplete risk factor assessment for household members that may underestimate risk factors, and ability to identify only coinsured household members, which may underestimate household size and overestimate the proportion of family members with risk factors.

Our study highlights that EHR data can be used to identify households at risk for diabetes. Health systems could use EHRs to screen family members for risk factors and support care coordination among families for cardiometabolic risk reduction. Family care coordination could reduce health system costs; however, few prevention programs enroll households at risk, reflecting a missed opportunity for population-level diabetes prevention.

## Notes

Supplement 1.
eMethods.

eReferences.

Supplement 2.
Data Sharing Statement
