# Supplementary material for: Identifying Household Diabetes Risk for Family Diabetes Prevention Using Electronic Health Records
Source: JAMA Netw Open. 2026 Jan 13;9(1):e2551823. doi: 10.1001/jamanetworkopen.2025.51823 (PMC12801083; doi:10.1001/jamanetworkopen.2025.51823)
Supplement: Supplement 1. — eMethods. eReferences. [file jamanetwopen-e2551823-s001.pdf]

Supplementary Online Content

Thomas TW, Finertie H, Silverberg A, et al. Identifying household diabetes risk for family diabetes prevention using electronic health records. *JAMA Netw Open.* 2026;9(1):e2551823. doi:10.1001/jamanetworkopen.2025.51823

eMethods.

eReferences.

This supplementary material has been provided by the authors to give readers additional information about their work.

eMethods.

Study design and participants

The study population was derived from Kaiser Permanente Northern California (KPNC), an integrated health delivery system with over 4.5 million members. The primary data source was the EHR system, which combines diagnosis, procedure, utilization, enrollment, and laboratory records from across the care system. We included KPNC health plan members who are 18-89 years of age and older, with a body mass index (BMI) of ≥25 (or ≥23 if Asian) and ≤ 70, who were insured through original Medi-Cal, managed Medi-Cal, the California Health Insurance Exchange, or commercially insured, and had evidence of prediabetes within 1 year prior to 1/1/2023 through 12/31/23. Individuals with type 1 or type 2 diabetes (other than gestational diabetes) or end-stage renal disease were excluded. These inclusion and exclusion criteria were based on eligibility requirements for enrollment in the National Diabetes Prevention Program. Those without continuous health plan coverage for 12 months prior to index date were excluded. Index date was the earliest of 1/1/2023 or first date inclusion criteria was met between 1/1/2023-12/31/2023.

Household members were those co-residing with and under the same insurance plan as the index participant. Co-residence was defined as living at the same address as the index participant at index date. We used the subscriber medical record number to link household members receiving insurance through the same subscriber, either as the subscriber or the dependent.

Study variables

Index cohort and household member characteristics including demographics, enrollment, and utilization data were pulled for the index cohort within 1-year before index date. We used a 2-year lookback for household member clinical factors, including lab results and primary care visits, and diabetes risk factors. We used a longer lookback period for household members because they were identified through the index person's care utilization and therefore may not have been as active in our health system. BMI for adults aged 20 and older was categorized according to established criteria. Age and sex-specific BMI for those 2-19 was calculated using the CDC's 2022 SAS program for CDC Growth Charts.[3] The most recent test results for both hemoglobin A1c and fasting plasma glucose were reported. If multiple results for the same test were collected in one day, the lowest result was selected. The total number of lab results of either type on separate days was included in the total number of glucose screening visits variable. We assessed diabetes status using the health system's diabetes registry. [4]

Our stratification of results by race and ethnicity was motivated by community partners who provide family-based diabetes prevention services. Race and ethnicity information was self-reported and collected via patient registration, available through the electronic health record and health insurance plan data. Patients self-reported race and ethnicity as Asian American, Black, Hawaiian or Pacific Islander, Hispanic or Latino, Multi-racial, Native American or American Indian, Unknown, or White.

Missingness for categorical variables was categorized as 'Missing' or 'Unknown'. Missingness for continuous variables was reported as N (percentage). Lack of labs results or diagnostic history in the EHR was coded as not present for indicator variables.

Statistical Analysis

Descriptive statistics were output as mean (standard deviation) for continuous variables and N (percentage) for categorical variables. For the index cohort, results were stratified by household composition and for individual household members, results were stratified by age.

© 2025 Thomas TW et al. *JAMA Network Open.*

eReferences.

1. Epstein LH, Wilfley DE, Egede LE. Transgenerational clinical care—The case for family-based treatment. JAMA Pediatr. 2025;179(2):120-121.
2. Aasbjerg K, Nørgaard CH, Vestergaard N, et al. Risk of diabetes among related and unrelated family members. Diabetes Res Clin Pract. 2020;160:107997.
3. Hales CM, Freedman DS, Akinbami L, Wei R, Ogden CL. Evaluation of alternative body mass index (BMI) metrics to monitor weight status in children and adolescents with extremely high BMI using CDC BMI-for-age growth charts. Published online 2022.
4. Moffet HH, Adler N, Schillinger D, et al. Cohort Profile: The Diabetes Study of Northern California (DISTANCE)—objectives and design of a survey follow-up study of social health disparities in a managed care population†. Int J Epidemiol. 2009;38(1):38-47. doi:10.1093/ije/dyn040

© 2025 Thomas TW et al. *JAMA Network Open.*
