# Supplementary material for: Identifying Household Diabetes Risk for Family Diabetes Prevention Using Electronic Health Records
Source: JAMA Netw Open. 2026 Jan 13;9(1):e2551823. doi: 10.1001/jamanetworkopen.2025.51823 (PMC12801083; doi:10.1001/jamanetworkopen.2025.51823)
Supplement: Supplement 2. — Data Sharing Statement [file jamanetwopen-e2551823-s002.pdf]

Data Sharing Statement

Thomas. Identifying Household Diabetes Risk for Family Diabetes Prevention Using Electronic Health Records. *JAMA Netw Open*. Published January 13, 2026.
doi:10.1001/jamanetworkopen.2025.51823

Data
Data available: No

Additional Information
Explanation for why data not available: The datasets generated during and/or analyzed during the current study are not publicly available due to patient data confidentiality.
